# Epidemiology and biology of cutaneous human papillomavirus

**DOI:** 10.6061/clinics/2018/e489s

**Published:** 2018-08-03

**Authors:** Emily M Nunes, Valéria Talpe-Nunes, Laura Sichero

**Affiliations:** Centro de Investigação Translacional em Oncologia, Instituto do Cancer do Estado de Sao Paulo (ICESP), Hospital das Clinicas HCFMUSP, Faculdade de Medicina, Universidade de Sao Paulo, Sao Paulo, SP, BR

**Keywords:** Human Papillomavirus, Cutaneous, Prevalence, Nonmelanoma Skin Cancer

## Abstract

Cutaneous human papillomaviruses (HPVs) include β- and γ-HPVs, in addition to a small fraction of α-HPVs. β-HPVs were first isolated from patients with the rare genetic disorder *Epidermodysplasia verruciformis*, and they are associated with the development of nonmelanoma skin cancer at sun-exposed skin sites in these individuals. Organ transplant recipients also have greater susceptibility to β-HPV infection of the skin and an increased risk of developing nonmelanoma skin cancer. In both immunosuppressed and immunocompromised individuals, cutaneous HPVs are ubiquitously disseminated throughout healthy skin and may be an intrinsic part of the commensal flora. Functional analysis of E6 and E7 proteins of specific cutaneous HPVs has provided a mechanistic comprehension of how these viruses may induce carcinogenesis. Nevertheless, additional research is crucial to better understand the pathological implications of the broad distribution of these HPVs.

## INTRODUCTION

**Figure 1:**
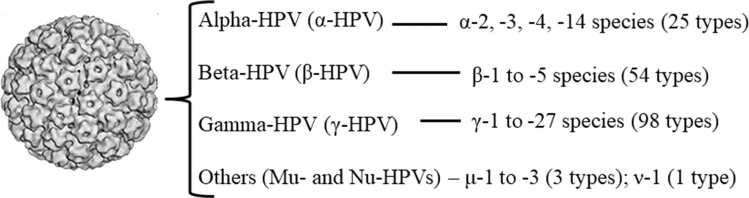
Distribution of cutaneous viral types within different HPV genera. The number of cutaneous viral types within each genus is indicated.

Human papillomavirus (HPV) represents a diverse group of viruses infecting mainly epithelial and mucosal tissues 1. Based on the identity of the *L1* major capsid gene sequence, the majority of the over 200 viral types characterized to date cluster within the alpha (α)-, beta (β)-, or gamma (γ)-HPV genus 2,3. While the great majority of α-HPVs are mucosal types isolated from the anogenital epithelia, some viral types in this genus (e.g., HPVs 2, 3 and 10) and β- and γ-HPVs were originally designated cutaneous types (Figure 1). To date, 54 β-HPVs (subdivided into 5 species, β1-5) and 98 γ-HPVs (subdivided into 27 species, γ1-27) have been fully sequenced and characterized (http://www.nordicehealth.se/hpvcenter/reference_clones/), and it is expected that these numbers will further increase once partial sequences of putative novel types are described 4. Some cutaneous HPVs are clearly associated with the development of various skin lesions, from warts to carcinomas, in restricted populations 1,5. Nevertheless, it has proven difficult to determine the role of particular β-HPVs in cutaneous malignancies because of the high viral diversity and ubiquity of multiple types throughout healthy skin, the oral cavity, the nasal mucosa and the anogenital region 6-11.

### Prevalence and distribution of cutaneous HPVs among immunosuppressed individuals

The first record concerning the association of HPV with papillomatous skin lesions that harbored carcinogenic potential dates to the early 1920s, when Lewandowsky and Lutz 12 first described a hereditary condition named *Epidermodysplasia verruciformis* (EV) that is characterized by extensive warts throughout the body (Table 1). Later, Jablonska et al. 13 observed that EV individuals infected with β-HPV 5 and 8 had a higher risk of developing nonmelanoma skin cancer (NMSC), particularly in ultraviolet (UV)-exposed sites. Together, both viral types are detected in approximately 90% of skin squamous cell carcinomas (SCCs) in EV patients. In these cases, β-HPVs are actively transcribed and generally persistent at high copy number 14. Currently, β-HPV 5 and 8 are accepted as possible etiological agents (carcinogen group 2B) of cutaneous SCC (cSCC) in immunosuppressed EV individuals by the International Agency for Research on Cancer (IARC) 15.

In the following years, several studies focused on analyzing the oncogenic potential of cutaneous HPVs in other immunosuppressed individuals, among which organ transplant recipients (OTRs) were the most extensively investigated. OTRs slightly resemble EV patients in that they are often covered with wart-like skin lesions and actinic keratosis (AK). Furthermore, OTRs have up to a 100-fold increased risk of developing NMSC compared to the general population 16-17. Importantly, the increased risk of NMSC is mostly associated with a higher incidence of cSCC 18-20. Clinical and histological features of these lesions suggest that cSCCs occasionally develop from viral warts or other precursor lesions 21,22. NMSC in OTRs often presents as multiple lesions and is usually confined to UV-exposed anatomical sites, most likely associated with local immunosuppression 23,24. These tumors are also more aggressive in OTRs than in the general population and form metastases more readily 18,25.

Among OTRs, cutaneous warts were detected in 43% of individuals at 3 months to 9 years following transplant 25,26. Additionally, within 15 years of transplantation, up to 90% of OTRs develop warts and/or cSCC 17. Upon analyzing skin smears from OTRs, dialysis patients and healthy controls, Antonsson et al. 6 observed that 11.5% of OTRs reported ever having skin cancer, whereas no cases of NMSC were observed in the other groups studied. NMSC incidence in OTRs varies depending on the duration of immunosuppression; Hardie et al. 18 demonstrated that the incidence of skin cancer increased 5% per year after the first year of transplant, with a cumulative risk of 44% after 9 years.

The incidence of NMSC is also related to long sun exposure. Boyle et al. 27 observed that 18% of renal transplant patients with high exposure levels to sunshine (>3 months in a tropical or subtropical climate or >5 years in an outdoor occupation) developed carcinogenic alterations in their skin: two patients were diagnosed with cSCC, and seven were diagnosed with AK, whereas neither lesion type was noted in the other patients or in the control group.

Although cumulative sun exposure is the major risk factor for NMSC, recent studies have revealed a role for HPV as a cofactor in association with UV radiation in cSCC in OTRs. OTRs have a higher cutaneous HPV prevalence rate in cSCC (up to 90%) than in normal skin (11-32%) 28. These infections frequently persist 29,30, and it has been observed that older age and a history of sunburn are associated with an elevated risk of persistent β-HPV infection 30,31. Furthermore, a significant association between the number of β-HPVs detected in eyebrow hair follicles and an increased risk of cSCC was reported among OTRs from Europe (the Netherlands, the United Kingdom, France and Italy) 32. Moreover, individuals with concordant β-HPV DNA in plucked eyebrow hairs and serologic tests had a significantly increased risk of developing SCC 33.

Among immunosuppressed OTRs, the occurrence of multiple cutaneous HPV infections is common 34, but high viral loads were shown to be associated with an increased risk of SCC development, with total load seemingly more important than the individual load of any specific type 32. It should be noted, however, that β-HPV is more highly prevalent in skin wart biopsies from OTRs than in either normal skin or plucked hairs from these patients 29,. Serological studies have also demonstrated that seroconversion to β-HPV increases with age 38,39 and have revealed a positive epidemiological association between β-HPV seroreactivity and cSCC development 40, even though not every infection is accompanied by a detectable or relevant seroresponse.

Nevertheless, given the high incidence of cSCCs in OTRs, identifying a clear link between β-HPV infection and cSCC would have important implications for therapy and prevention 41,42. Therefore, more recent case-control studies are ongoing, focusing on the association of cutaneous HPV in the early stages of NMSC carcinogenesis in immunosuppressed individuals. However, the data are inconclusive 42-46.

### Prevalence and distribution of cutaneous HPVs among immunocompetent individuals

Cutaneous β- and γ-HPV DNA can be detected beginning in early infancy and may be detected in 70% of children by 4 years of age 47. Additionally, β-HPV types detected on parents are more commonly found on their babies 47,48. Viral transmission seems to occur inevitably through direct skin contact 10,, and these viruses have been suggested to be commensal to humans 38,52,53 (Table 2).

β-HPVs are widespread in immunocompetent (IC) individuals within the general population: when plucked hairs from different body sites are tested, the prevalence is approximately 90% 54,55. It is believed that cutaneous HPVs target the hair follicle bulge, which is probably the reservoir of these viruses 32. Therefore, eyebrow hairs have served as an easily obtained material for marker analysis in several epidemiological studies and seem to reflect infection in other parts of the body 35.

Advanced age has been shown to be the most important factor influencing the presence of β-HPV DNA in IC individuals 56,57. Furthermore, sun exposure and a history of skin cancer are risk factors associated with β-HPV detection in these individuals 6. For OTRs, some studies have investigated the prevalence of cutaneous HPVs among IC individuals of different ethnicities and residing under different climate conditions 52. It was reported that the prevalence of HPV DNA was lower in samples from Zambia than in those collected in Sweden (*p*<0.01) and Bangladesh (*p*<0.05) 52. β-HPV prevalence and distribution studies have shown that viral positivity was, on average, higher on the forehead (36%) and back of the hand (38%) than on the buttocks (26%), indicating that UV radiation may be a putative risk factor for viral infection, even though sun exposure data were not collected in this specific study 48. In fact, severe sunburns have been associated with the presence of β-HPV DNA 24. Second-degree burns and repetitive sunburns, with skin regeneration of the underlying capillary bulb, may result in the amplification of β-HPV DNA by activating the HPV life cycle 24. In fact, the risk for SCC development among Australian or Netherlander IC individuals is higher for those in which β-HPV DNA was detected at high loads in plucked eyebrow hairs 32,56,58.

β-HPV persistence was more commonly observed in adults (92%) than in children (66%), and although multiple β-HPVs can infect persistently, no specific type seems to predominate in such infections 48. Nevertheless, it must be highlighted that the prevalence of β-HPVs significantly decreases after tape stripping, indicating that only a small number of epithelial cells are in fact infected, and most of the detected viruses may reflect deposition throughout the external skin surface 23.

The IARC recognizes the need for further research on cutaneous HPVs to better understand the widespread distribution of these viruses. β-HPVs may also play a role in the pathogeny of NMSC in healthy individuals 59; however, to date, epidemiological evidence is inconclusive concerning the association between specific β- and γ-HPVs and the development of skin cancer in IC individuals 14,15.

Regarding the HPV status in NMSCs from immunosuppressed and IC individuals, viral prevalence was higher in the former group for all lesion types analyzed: premalignant lesions (88% among immunosuppressed *vs* 54% among IC), SCC (84% *vs* 27%) and basal cell carcinoma (BCC, 75% *vs* 36%) 36. Nevertheless, the prevalence and spectrum of HPV types detected within the two populations were equivalent among premalignant lesions, SCC and BCC, and HPVs 5 and 8 were the most frequently identified types 60. Interestingly, β-HPV prevalence was reported to be higher in premalignant AK than in cSCC, and real-time PCR analysis indicated higher viral loads in premalignant lesions 46 than in SCC, in which viral load rarely reaches the level of one viral copy per cell 43,46. This scenario is compatible with a carcinogenic role for HPV at the early stages of skin carcinogenesis. Because cSCC most commonly develops in sun-exposed anatomical sites, it is reasonable to suppose that UV radiation may impede HPV antigen presentation by suppressing local cell immunity 61. In addition, several studies have suggested the importance of β-HPVs as cofactors to UV radiation in the development of SCC by facilitating the accumulation of UV-induced mutations, which can ultimately lead to cell transformation.

In addition to HPV DNA detection, the detection of antibodies to β-HPVs and their association with SCC risk development have been evaluated in several studies 44. Nevertheless, overall, serological studies show considerable heterogeneity in the results: whereas high overall seropositivity (>90%) to at least one viral β-HPV has been described 38,62, a lower prevalence is observed in other studies 33,63. The divergence in the data obtained in these studies could be attributed not only to differences in serological methods but also to the range of cutaneous HPVs tested. With β-HPV DNA detection, the seroprevalence has been shown to increase with age 6,30,55. Notably, β-HPV types most commonly detected in the skin have the highest seroprevalence worldwide 33.

Due to the wide distribution of cutaneous HPVs in the skin, several groups, including ours, have recently focused on investigating the distribution of cutaneous HPVs in other anatomical sites, including the anogenital area. Within the HIM (HPV Infection in Men) cohort study 64, we initially observed that most of the ∼15% of male genital samples that could not be classified with widely used α-HPV typing technologies harbored β- and γ-HPVs, as evidenced by using a PCR sequencing protocol 65. We further observed that most samples were positive for HPV DNA of multiple cutaneous types using a sensitive Luminex-based methodology, suggesting that the former protocol could underestimate the true prevalence of cutaneous β- and γ-HPVs in the male genital region 65,66. In order to better understand the prevalence and distribution of cutaneous HPVs, we and others further analyzed β- and γ-HPV DNA and antibodies by Luminex methodology in a series of samples obtained from the anogenital region of both men and women 9,51,, the oral cavity 7,9,70, the skin 31, and the nasal cavity 8. Taken together, these studies corroborate that cutaneous HPVs are ubiquitously disseminated throughout healthy skin and may be an intrinsic part of the commensal flora.

We further observed that male external genital lesions (EGLs) are not associated with β-HPV infections 68,71 and that the detection of DNA from these viruses is not associated with sexual risk factors, indicating other routes of transmission, such as autoinoculation and nonpenetrative sexual activities 9,65-67,. Alternatively, the detection of β-HPVs at one anatomic site may indicate the deposition of virions shed from other anatomic sites 50,65,66,69. Nevertheless, it was recently reported that among heterosexual couples, the transmission rate of β-HPVs between anogenital sites was 15.9 per 100 person-months from men-to-women, with a similar risk for women-to-men transmission, suggesting that β-HPVs can be sexually transmitted 51. Lastly, the few reports in which the oral and anogenital regions were analyzed concurrently indicated that simultaneous oral-genital type-specific β-HPV infections are relatively rare 10, but seem to be higher across keratinized tissues than across mucosal sites 74. Nevertheless, given the large number of samples and β-HPV types analyzed, it is unlikely that the same HPV type will be found at a distant skin site by chance.

### Functional analysis of cutaneous HPVs

HPVs are small, nonenveloped viruses with circular double-stranded DNA of approximately 8000 bp. The viral genome is divided into three regions: the long control region (LCR) contains cis-responsive elements for viral and cellular proteins that regulate viral gene expression and replication; the early region (E), which encodes proteins crucial to viral transcription and replication; and the late region (L), which encodes the viral capsid structural proteins 78-80. Although the viral genome structure and organization are highly conserved among HPVs, the LCR of β-HPVs is shorter than that of α-HPVs, and the E5 gene is absent from the β-HPV genome 81-83.

The HPV life cycle is tightly associated with the differentiation of the stratified squamous epithelium. HPV infection begins with entrance of the virus into the basal layer of the epithelia due to microtrauma 79,84,85. At least for high-risk α-HPV-infected tissues, the differentiation process is altered by expression of the E6 and E7 viral oncoproteins, which interact principally with the TP53 and pRb suppressor proteins, respectively, but also interact with a broad spectrum of other cellular proteins, altering the biological properties of the host cell 79,83,.

As previously discussed in this review, β-HPVs most likely play a role in the initiation of cSCC rather than in the maintenance of the transformed phenotype 14. It is hypothesized that β-HPV infections destabilize the host genome, allowing tumors to further develop in the absence of the viral genome 90-92. Some studies have provided a mechanistic comprehension of how these viruses induce carcinogenesis and have indicated that the biology involved in β-HPV-mediated skin carcinogenesis differs from that induced by high-risk α-HPVs 80,91,93,94.

**Figure 2:**
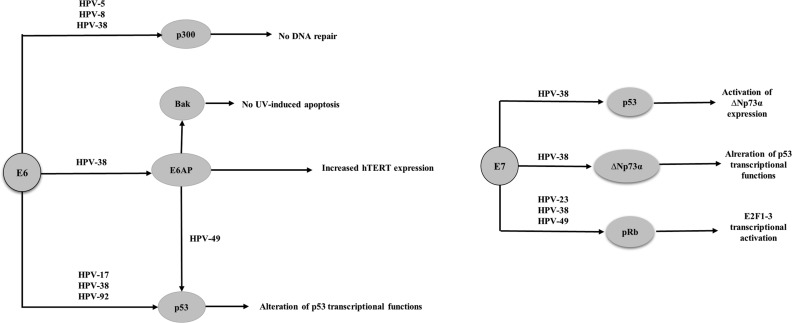
Cellular targets of the E6 and E7 proteins from specific β-HPVs.

Studies have shown that β-HPVs 38 and 49 are able to immortalize primary human keratinocytes, whereas HPVs 10, 14, 22, 23, 24 and 36 do not have this ability 95-97. It has also been reported that transgenic mice expressing the HPV 38 E6 and E7 proteins under control of a keratinocyte-specific promoter exhibit epidermal hyperplasia and are susceptible to the development of cutaneous tumors promoted by chemical carcinogens and UV radiation 98-101. Although the E6 protein from HPVs 8, 24 and 38 binds *in vitro* to E6-AP (E6-associated protein), p53 degradation was observed in the presence of only HPV 49 95-97, (Figure 2). The E6 protein from β-HPVs 5, 8, and 38 attenuates p53 phosphorylation and ubiquitination in response to UV exposure, resulting in less efficient repair of damaged cellular DNA 90,96. Additionally, HPV 38 induces telomerase by a mechanism dependent on E6-AP 95. HPV 38 E6 also alters the capacity of p53 to activate proteins involved in apoptosis and suppress proliferation by inducing the accumulation of ΔNp73, a p53 isoform that antagonizes p53 96. The E6 protein from HPVs 5, 8 and 38 was shown to bind p300, preventing p53 acetylation and p53 -induced repair and transcriptional transactivation, thus contributing to the accumulation of mutations and chromosomal abnormalities 90. Furthermore, as with α-HPV, the E6 protein of some β-HPVs induces BAK degradation, thus preventing the release of pro-apoptotic mitochondrial factors 103. The interaction of E6 with E6-AP is required not only for BAK degradation but also for hTERT (human telomerase reverse transcriptase) induction 104,105.

The E7 proteins from the cutaneous HPVs 154, 22, 23, 24, 36, 38 and 49 bind *in vitro* to pRb but are unable to induce pRb degradation when expressed in human keratinocytes 97, (Figure 2). Nevertheless, in human keratinocytes transduced with HPV 38 and 39 E6 and E7 proteins, E2F-induced transcription is likely activated because these viral proteins induce pRb hyperphosphorylation 80,95,97.

Although most research on oncogenic potential and disease association has focused on α-HPVs, there is interest in identifying a role of non-α HPV types in the pathogenesis of benign and malignant lesions (Table 3). Challenges in finding relevant associations between cutaneous HPV infection and NMSC development include the multiplicity and ubiquity of these viruses throughout the human body, the high probability of viral transmission (including autoinoculation), and differences observed in the carcinogenic potential of individual β-HPVs (Table 4). The last IARC monograph (100B) was unable to identify consistent epidemiological evidence for an etiological role attributable to any specific cutaneous HPV type or species in NMSC development. In addition, biological mechanisms explaining the oncogenicity of these viruses have not been fully elucidated.

## AUTHORS’ CONTRIBUTIONS

Nunes EM, Talpe-Nunes V and Sichero L critically discussed and wrote the manuscript.

## Figures and Tables

**Table 1:** Select studies on the prevalence and distribution of cutaneous HPVs among immunosuppressed individuals.

Year	Author (s) (reference number)	Data
1922	Lewandowsky and Lutz 12	First description of epidermodysplasia verruciformis (EV).
1972	Jablonska et al. 13	β-HPVs 5 and 8 infected EV individuals had a higher risk of developing NMSC (after UV exposition).
1974	Koranda et al. 26	Cutaneous warts were detected in 43% of ORT individuals after 3 months to 9 years following transplant.
1976	Mullen et al. 20	Increased risk of NMSC is mostly associated to higher incidence of cSCC.
1977	Hoxtell et al. 19	
1980	Hardie et al. 18	
1978	Sbano et al. 22	cSCC occasionally develop from viral warts or other precursor lesions.
1989	Barr et al. 21	
1980	Hardie et al. 18	The incidence of skin cancer increases 5% per year after the first year of transplant, with a cumulative risk of 44% after 9 years.
1980	Hardie et al. 18	Tumors are more aggressive in OTRs than in the general population.
1984	Boyle et al. 27	18% of renal transplant patients who were highly exposed to UV developed carcinogenic lesion in the skin.
1995	Birkeland et al. 16	OTRs have until 100 fold increased risk of developing NMSC as compared to the general population.
2000	Lindelöf et al. 17	
1997	Boxman et al. 35	β-HPV is more prevalent in skin warts biopsies than in both the normal skin and plucked hairs among OTRs.
2000	Harwood et al. 36	
2003	Meyer et al. 37	
2000	Antonsson et al. 6	Among OTRs, dialysis patients, and healthy controls, solely the first group reported ever having skin cancer (11.5%).
2000	Lindelöf et al. 17	Within 15 years of transplantation, up to 90% of OTRs develop warts and/or cSCC.
2000	Berkhout et al. 29	Infections of cutaneous HPVs frequently persist in OTRs.
2007	Hazard et al. 30	
2003	Feltkamp et al. 40	There is a positive epidemiological association between β-HPV seroreactivity and cSCC development.
2004	Termorshuizen et al. 24	NMSCs among OTRs are often multiple and usually confined to UV-exposed anatomical sites.
2004	Harwood et al. 34	
2007	Forslund et al. 23	
2005	Weissenborn et al. 46	Data regarding the association between cutaneous HPV infection and cSCC is still inconclusive.
2008	Rollison et al. 45	
2011	Arron et al. 43	
2016	Chahoud et al. 44	
2007	Nindl et al. 28	OTRs have higher cutaneous HPV prevalence rate up to 90% in cSCC compared to the normal skin (11-32%).
2007	Hazard et al. 30	Older ages and history of sunburn are associated to an elevated risk of β-HPV persistent infection.
2014	Hampras et al. 31	
2008	Michael et al. 39	Seroconversion to β-HPV increases with age.
2010	Antonsson et al. 38	
2009	Bouvard et al. 15	β-HPVs 5 and 8 are accepted as possible etiological agents (carcinogens group 2B) of cSCC in immunosuppressed EV individuals.
2011	Proby et al. 33	Individuals with concordant β-HPV DNA in plucked eyebrow hairs and serologic tests had a significantly increased risk of developing SCC.
2013	Neale et al. 32	There is a significant association between the number of β-HPVs detected at eyebrow hair follicles and the increased risk of cSCC among OTRs.

**Table 2:** Select studies on the prevalence and distribution of cutaneous HPVs among immunocompetent individuals.

Year	Author (s) (reference number)	Data
1997	Boxman et al. 35	Cutaneous HPVs detection in eyebrow hairs seems to reflect infections in other parts of the body (useful in epidemiological studies).
2000	Antonsson et al. 6	Sun exposure and history of skin cancer are risk factors associated to β-HPVs detection in IC individuals.
2000	Harwood et al. 36	Cutaneous HPVs prevalence was higher among individuals who reported ever having skin lesions.
2000	Antonsson et al. 6	β-HPV DNA detection and seroprevalence increases with age.
2003	Struijk et al. 56	
2007	Hazard et al. 30	
2009	Weissenborn et al. 48	
2009	de Koning et al. 55	
2003	Antonsson et al. 47	The presence of cutaneous β- and γ-HPVs DNA is observed since early infancy.
2003	Antonsson et al. 47	β-HPVs types detected on parents are also more commonly found in their babies.
2009	Weissenborn et al. 48	
2003	Antonsson et al. 52	β- and γ-HPVs may be commensal to humans.
2010	Antonsson et al. 38	
2014	Bzhalava et al. 53	
2004	Termorshuizen et al. 24	Severe sunburns are associated with the presence of β-HPV DNA.
2004	Stockfleth et al. 60	HPVs 5 and 8 were the most frequently found in premalignant lesions, SCC and BCC.
2004	Smith et al. 75	Simultaneous oral-genital type-specific β-HPV infections are relatively rare.
2006	Fakhry et al. 73	
2011	Termine et al. 77	
2017	Hampras et al. 74	
2017	Steinau et al. 76	
2017	Nunes et al. 10	
2005	Weissenborn et al. 46	Higher viral loads are detected within pre-malignant skin lesions as compared to SCC.
2011	Arron et al. 43	
2007	Forslund et al. 23	Most viruses detected on the external skin surface may reflect HPV deposition.
2007	Köhler et al. 54	The prevalence of β-HPVs DNA in plucked hairs from different body sites of IC individuals is approximatelly 90%.
2009	de Koning et al. 55	
2008	Patel et al. 59	β-HPVs may play a role in the pathogeny of NMSC also in healthy individuals.
2008	Feltkamp et al. 49	Viral transmission seems to occur through direct skin contact.
2017	Moscicki et al. 51	
2017	Nunes et al. 10	
2009	Bouvard et al. 15	Epidemiological evidence concerning the association between specific β- and γ-HPVs and the development of skin cancer in IC is inconclusive.
2009	Weissenborn et al. 48	UV radiation may be a putative viral detection-related risk factor.
2010	Antonsson et al. 38	High overall seropositivity (>90%) to at least one viral β-HPV is observed within healthy individuals.
2010	Iannacone et al. 62	
2010	Michael et al. 63	Low overall seropositivity to at least one viral β-HPV is observed within healthy individuals.
2011	Proby et al. 33	
2011	Proby et al. 33	β-HPV types most commonly detected in the skin also have the highest seroprevalence.
2011	Bottalico et al. 7	Analysis of β- and γ-HPVs DNA and antibodies prevalence among series of samples (anogenital, oral, skin, nasal cavity from women and men).
2013	Forslund et al. 8	
2013	Pierce Campbell et al. 68	
2013	Sichero et al. 65	
2013	Paolini et al. 70	
2014	Hampras et al. 31	
2014	Sichero et al. 66	
2015	Sichero et al. 69	
2015	Donè et al. 67	
2016	Nunes et al. 9	
2017	Moscicki et al. 51	
2013	Neale et al. 32	Cutaneous HPVs target the hair follicle bulge, which is probably the reservoir of these viruses.
2013	Sichero et al. 65	The majority of male genital samples could not be classified using technologies widely used for typing of α-HPVs.
2013	Pierce Campbell et al. 68	Male external genital lesions (EGL) are not associated to β-HPVs infections.
2016	Rahman et al. 71	
2013	Sichero et al. 65	Most samples from the male genitals were positive for multiples cutaneous HPV DNA.
2014	Sichero et al. 66	
2013	Sichero et al. 65	The detection of β-HPVs in one anatomic site may also represent deposition of virions shed from other anatomic sites.
2014	Sichero et al. 66	
2015	Sichero et al. 69	
2013	Sichero et al. 65	The detection of cutaneous HPVs DNA is not associated to sexual risk factors. Other routes of transmission such as autoinoculation and non-penetrative sexual activities could be associated.
2014	Sichero et al. 66	
2015	Donè et al. 67	
2015	Torres et al. 72	
2016	Nunes et al. 9	
2016	Chahoud et al. 44	Analyses of association between the detection of antibodies to β-HPVs with SCC risk development.
2017	Hampras et al. 74	The occurrence of concordant β-HPV infections seem to be higher across keratinized tissues than across mucosal sites.
2017	Moscicki et al. 51	The transmission rate of β-HPVs between anogenital sites from men-to-women and women-to-men was similar, suggesting these are sexually transmitted.

**Table 3:** Highlights regarding the epidemiology and biology of cutaneous human papillomavirus in immunosuppressed and immunocompetent individuals.

• β- and γ-HPVs, in addition to few α-HPVs are originally designated “cutaneous types”.
• The IARC classified β-HPVs 5 and 8 as possible etiological agents of skin SCC in EV individuals.
• OTRs not only have a higher susceptibility to β-HPVs but further attain an ~100 fold increased risk of developing NMSC as compared to the general population.
• Among OTRs and IC individuals cutaneous HPVs are ubiquitously spread throughout the body and may be an intrinsic part of the commensal flora.
• Although cumulative sun exposure is the major risk factor for NMSC, studies points towards β-HPV infections as co-factors in skin SCC in association with UV radiation.
• β-HPVs most probably play a role in the initiation of skin SCC rather than in the maintenance of the transformed phenotype.
• Function analysis of E6 and E7 proteins of specific cutaneous HPVs indicate that the biology involved in β-HPV mediated skin carcinogenesis differ from that induced by high-risk α-HPV types.

**Table 4:** Challenges/critical open questions regarding the epidemiology and biology of cutaneous human papillomavirus in immunosuppressed and immunocompetent individuals.

• Are cutaneous HPVs associated to the development of non-melanoma skin cancer among immunosuppressed and immunocompetent individuals?
• Why are cutaneous HPVs more diverse than their mucosal counterparts?
• Do cutaneous HPVs contribute to carcinogenesis associated to other carcinogens at skin and non-skin sites?
• Do cutaneous HPVs contribute to high-risk mucosal carcinogenesis in cases of co-infections?
• Why are cutaneous HPVs more prevalent in precursor lesions compared to malignant lesions?
• Why do specific β-HPVs deregulate fundamental cellular events intimately linked to transformation *in vitro*, but do not cause cancer in humans?
